# A Comparative Genomics Study on the Molecular Evolution of Serotonin/Melatonin Biosynthesizing Enzymes in Vertebrates

**DOI:** 10.3389/fmolb.2020.00011

**Published:** 2020-02-04

**Authors:** Yunyun Lv, Yanping Li, Jia Li, Chao Bian, Chuanjie Qin, Qiong Shi

**Affiliations:** ^1^Key Laboratory of Sichuan Province for Fishes Conservation and Utilization in the Upper Reaches of the Yangtze River, College of Life Sciences, Neijiang Normal University, Neijiang, China; ^2^Shenzhen Key Lab of Marine Genomics, Guangdong Provincial Key Lab of Molecular Breeding in Marine Economic Animals, BGI Academy of Marine Sciences, BGI Marine, BGI, Shenzhen, China

**Keywords:** serotonin, melatonin, biosynthesizing enzyme, circadian rhythm, molecular evolution, vertebrate

## Abstract

Serotonin is important in vertebrates for its crucial roles in regulation of various physiological functions. Investigations on how the biosynthesizing enzymes mediate serotonin production and conversion during biological processes have been active in the past decades. However, a clear-cut picture of these enzymes in molecular evolution is very limited, particularly when the complexity is imaginable in fishes since teleosts had experienced additional whole genome duplication (WGD) event(s) than tetrapods. Since serotonin is the main intermediate product during melatonin biosynthesis from tryptophan, we therefore summarize an overview of recent discoveries about molecular evolution of the four melatonin biosynthesizing enzymes, especially the L-aromatic amino acid decarboxylase (AAAD) for serotonin production and aralkylamine N-acetyltransferase (AANAT) for serotonin conversion in vertebrates. Novel copies of these genes, possibly due to WGD, were discovered in fishes. Detailed sequence comparisons revealed various variant sites in these newly identified genes, suggesting functional changes from the conventional recognition of these enzymes. These interesting advances will benefit readers to obtain new insights into related genomic differences between mammals and fishes, with an emphasis on the potential specificity for AANAT in naturally cave-restricted and deep-sea fishes.

## Introduction

Serotonin (5-hydroxytryptamine, 5-HT) acts as a critical neurotransmitter in the central nervous system (CNS) and an important hormone in the peripheral tissues (Keszthelyi et al., 2010). Although it is unable to go through the blood-brain barrier, serotonin contents in the two pools are different, of which 95% is produced in the peripheral tissues and only 5% in the brain (El-Merahbi et al., 2016). Unlike many classical hormones that usually distribute in limited tissues, serotonin can be widely traced in various anatomical organizations. Interestingly, serotonin is presented not only in vertebrates, but also in fungi, plants, and invertebrates (Srinivasan et al., 2008; Kang et al., 2009; Curran, 2012), suggesting an ancient evolutionary origin in the biological world. As one of crucial monoamines, the brain-derived serotonin involves multiple physiological processes such as behavior, learning, appetite, and glucose homeostasis (Marston et al., 2011; Klaus-Peter et al., 2012); however, the peripheral serotonin plays as a hormone to regulate various physiological functions along with blood circulation (Stunes et al., 2011; Yasmine et al., 2012).

**Figure 1:**
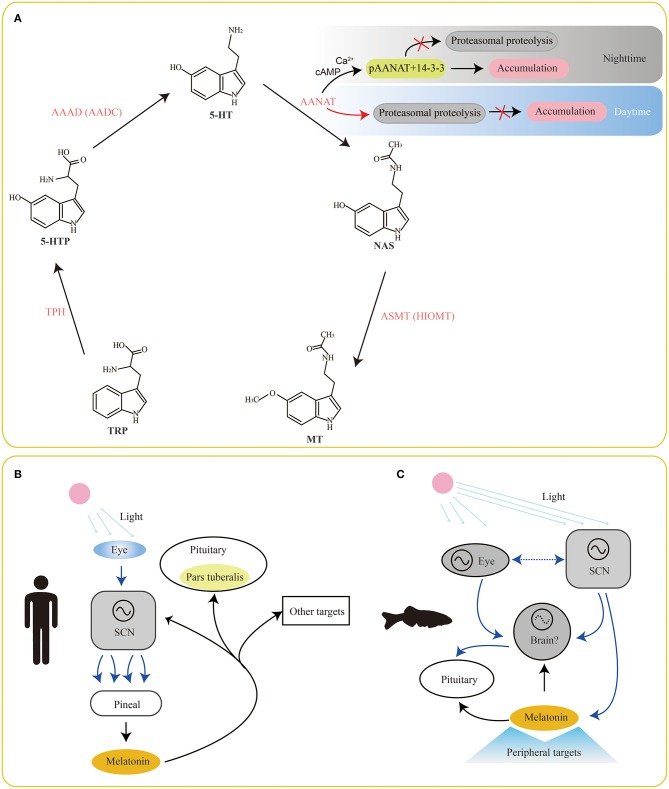
Involvement of serotonin/melatonin in the synchronization between environmental photoperiods and vertebrate circadian rhythms. The classical pathway of melatonin biosynthesis in vertebrates is summarized in **(A)**. Meanwhile, melatonin presents a feedback to the SCN, pituitary (pars tuberalis; **B**) and other brain areas for physiologic adjustments. However, this system is more complex in fishes **(C)** since their pineal organs are also photoreceptive. Find the detailed abbreviations of enzymes and molecules in corresponding texts.

Serotonin is the main intermediate product during melatonin biosynthesis from tryptophan (Figure 1A). Tryptophan (Trp) in cells is firstly hydroxylated by tryptophan hydroxylase (TPH) and then decarboxylated by L-aromatic amino acid decarboxylase (AAAD) to produce serotonin (Keszthelyi et al., 2010). However, synthesis of the periphery- and the brain-derived serotonin relies on two different TPH enzymes (TPH1 and TPH2, respectively). Subsequently, serotonin is catalyzed by the major rate-limiting enzyme aralkylamine N-acetyltranserase (AANAT) to generate N-acetylerotonin (NAS). Finally, NAS is transformed to melatonin via acetylserotonin O-methyltranserase (ASMT).

Obviously, serotonin locates in the center of the melatonin biosynthesis pathway. Therefore, understanding of related enzymes for melatonin synthesis is important for improving the recognition of serotonin functions. As we know, melatonin is mainly produced and secreted in the vertebrate pineal organs at night. This rhythmic pattern leads to a corresponding rhythmic melatonin levels in blood and cerebrospinal fluid during day and night. However, organization of the circadian system in relation to melatonin secretion is different between mammals and fishes (Figures 1B,C). In mammals, a primary linear route starts at reception of environmental photic signals in eyes (retinae), which are transmitted to the suprachiasmatic nuclei (SCN) of the hypothalamus via a retino-hypothalalamic tract (RHT). Since then, a multisynaptic pathway, comprised of SCN, preganglionic neurons of the sympathetic nervous system, superior cervical ganglion (SCG) and pineal organ (Figure 1B), is interconnected (Simonneaux and Ribelayga, 2003). This system is more complicated in fishes (Figure 1C), since their pineal organs are also photoreceptive (Falcón et al., 2007).

The nocturnal increase of melatonin at night mostly depends on the activity of AANAT by the synthesis pathway of serotonin→ ANNAT→ melatonin (Ganguly et al., 2005). Changes of AANAT activity are usually strongly controlled by cyclic AMP (cAMP)-dependent binding to dimeric 14-3-3 proteins. During nighttime, two sites (T31 and S205) occurring phosphorylation (to be pAANAT) prompt the binding between 14-3-3 proteins and pAANAT (Figure 1A). This action can protect the AANAT against proteasomal proteolysis and lead to a continuous accumulation of AANAT for 5-HT conversion and melatonin synthesis (Gastel et al., 1998; Obsil et al., 2001). Meanwhile, the binding to 14-3-3s also elevates the affinity of AANAT by ~10-fold for arylalkylamine substrates, such as serotonin and tryptamine (Ganguly et al., 2001). At daytime, decrease of cAMP levels results in dephosphorylation of pAANAT, and thereby the binding between 14-3-3s and pAANAT is broken out. This action prevents from protection of AANAT. Thus, the proteasomal proteolysis of AANAT at daytime stops the production of melatonin (Ganguly et al., 2001, 2005). This circle of phosphorylation and dephosphorylation of AANAT mainly contributes to the variation of melatonin production in a circadian rhythm.

In the past decades, various studies associated with serotonin and melatonin have been developed rapidly. Multifunction of serotonin as well as melatonin in various vertebrate lineages is hence clearer and clearer. However, species diversity is far more complex than what we expected. Certain species owing a special habitat, such as deep-sea or caved environment (dim or without lights) may lose most of their rhythmicity. They are thereby good models for examination of the relationships between molecular mechanisms and melatonin secretion, especially for understanding of serotonin synthesis related enzymes. It was reported that teleosts had experienced one more whole genome duplication (WGD; i.e., teleost-specific genome duplication, TSGD) at about 320 million years ago (Mya; Taylor et al., 2003; Jaillon et al., 2004) in comparison to tetrapods, adding much complexity to the serotonin/melatonin biosynthesizing enzymes. Here, we provide an overview to summarize recent discoveries about molecular evolution of the four biosynthesizing enzyme genes (especially the *aaad* for serotonin production and *aanat* for serotonin conversion) in vertebrates, with a focus on genomic differences between mammals and fishes.

## Serotonin-Related Genes Within the Melatonin Biosynthesis Pathway

### Tryptophan Hydroxylase (TPH)

TPH is the initial enzyme for melatonin biosynthesis (Figure 1A), transforming Trp into 5-hydroxytryptophan (5-HTP). It belongs to the superfamily of aromatic amino acid hydroxylase (McKinney et al., 2001). For over one decade, researchers had initially thought TPH to be with only one member before the study of Walther et al. (2003), in which the *tph* gene (now called *tph1*) was knocked out in mice. Surprisingly, the *tph1*-deficient mice lacked serotonin in the pineal gland and certain peripheral tissues (Walther et al., 2003). In addition, the serotonin content was observed to be slightly reduced in the brainstem of the *tph1*-deficient mice in comparison to the wild type, suggesting existence of another *tph* member that plays a compensatory role to *thp1*. Subsequently, these scientists verified a novel gene member of the TPH family and named it as *tph2*; the classical *tph* gene was since then renamed as *tph1* (Mockus and Vrana, 1998).

It has been demonstrated that *tph1* is mainly distributed in the pineal gland and peripheral gut, spleen, and thymus; *tph2* is predominantly expressed in the CNS, such as the brainstem (Cornide-Petronio et al., 2013). There are two independent serotonin systems in vertebrates that are regulated by the two different TPH enzymes with distinct functions as follows: *tph1* usually plays important roles in peripheral effects such as hemostasis, immune, melatonin synthesis, migraine, and vasoconstriction; *tph2* is often involved in effects of the CNS, such as aggression, anxiety, depression, epilepsy, food intake, and sleep. Fish is the most diverse vertebrate taxa; serotonergic neurons can be identified based on corresponding 5-HT levels, and thereby *tph* can be used as a specific marker for 5-HT generation (Xu et al., 2019).

**Figure 2:**
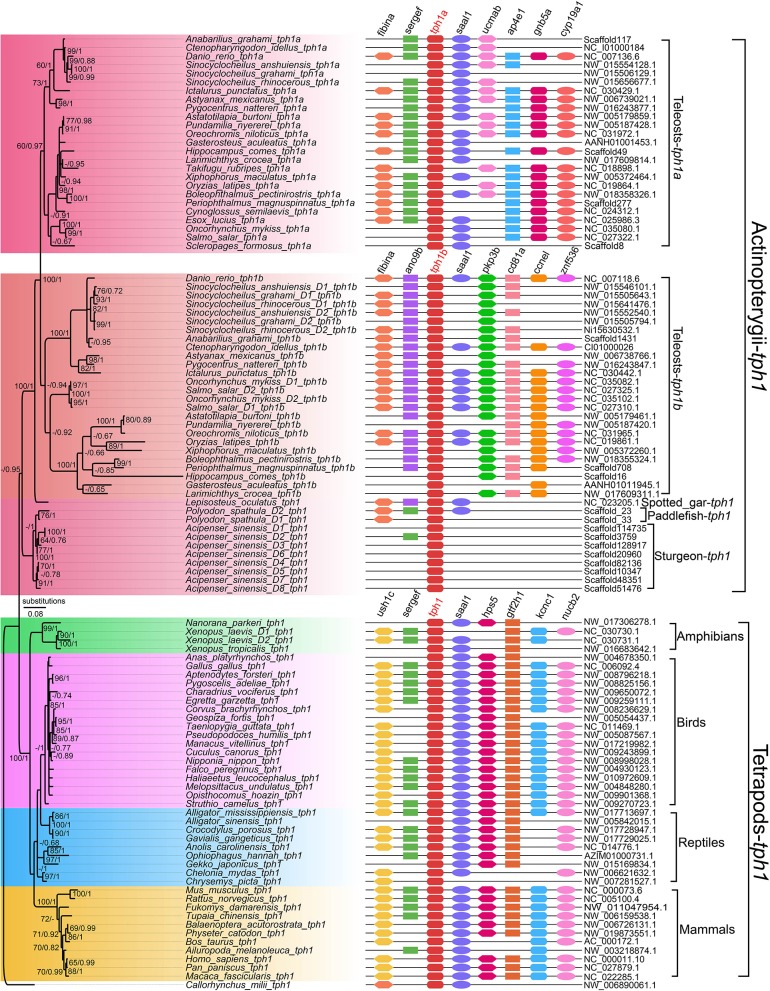
A Phylogenetic tree and genome synteny of *tph1* genes in vertebrates (adapted from Xu et al., 2019). The phylogenetic tree **(Left)** was constructed from 105 TPH1 protein sequences, with corresponding synteny data **(Right)** for validation.

More *tph* copies (*tph1a, tph1b*, and *tph2*) have been discovered in teleost species such as zebrafish, sticklebacks, and medaka (Bellipanni et al., 2002; Lillesaar, 2011). In our recent genomic investigation (Xu et al., 2019), we found that tetrapods and non-teleosts had two *tph* isotypes (*tph1* and *tph2*); however, in the teleost lineages, *tph1* was further diverged into *tph1a* and *tph1b*. Based on a detailed phylogenetic analysis (Figure 2), we propose that the TSGD events may have contributed to the division of three *tph* isotypes (*tph1a, tph1b*, and *tph2*). In addition, the *tph* copy numbers between diploid and tetraploid fish species are not always corresponding to a 1:2 (see more details in Xu et al., 2019); therefore, we infer that WGD and gene loss may have generated variations in *tph* gene copy numbers.

Certain important sites in representative TPH proteins were examined for structural comparisons, and we determined that the differences of vertebrate TPHs mainly located at the NH_2_-terminal (Xu et al., 2019). Predicted 3D structures between TPH1 and TPH2 revealed obvious variations in structural elements (α-helix or β-sheets) and loops.

Interestingly, here we also performed an in-depth investigation on *tph* isotypes in a cavefish Mexican tetra (*Astyanax mexicanus*), and found missing of *tph1b* in its genome. Although the loss of *tph1b* gene needs further validation, our preliminary data provide novel insights into *tph* evolution in vertebrates.

### L-Aromatic Amino Acid Decarboxylase (AAAD)

AAAD is for the second reaction, decarboxylating from 5-HTP to 5-HT, in melatonin synthesis (Figure 1A). It can also convert L-Dopa to dopamine (thereby named as DOPA decarboxylase, DDC). Due to its vital responsibility to synthesize these neurotransmitters, AAAD has been considered as an important decarboxylizing enzyme (Swoboda et al., 2003). Patients with AAAD deficiency presented compromised development, especially in motion activities (Hwu et al., 2012).

Moreover, AAAD is reported to be involved in many neurological diseases, such as Parkinson's disease and depression. When patients are treated with L-DOPA or 5-HTP, AAAD has become the critical rate-limiting protein in the synthetic pathways for both 5-HT and dopamine (Hwu et al., 2012). Studies on how AAAD participates in Parkinson's disease have been very active in mammals (Boomsma et al., 1989; Brun et al., 2010; Hwu et al., 2012). Related reports suggest that, via controlling the availability of 5-HT, AAAD may regulate melatonin synthesis (Adamska et al., 2016).

Recently, one of our genomic investigations indicated that tetrapods and diploid bony fishes had one *aaad* gene and a new *aaad*-like gene, which formed a novel AAAD family (Li et al., 2018). These novel *aaad*-like genes display high similarities to the *aaad* genes, while variations are also presented in the sequence alignments, especially the critical sites 298 and 302 (based on the human template of 3RBF; Giardina et al., 2011) were embedded in a heptapeotide region that may be acting as a cofactor binding site (Ichinose et al., 1989). The two sites may shift the protein structure, since one participates in the formation of α-helix and the another for β-strand. Hence, these newly identified *aaad*-like genes may play different functions due to their structural variations from the *aaad* genes.

**Figure 3:**
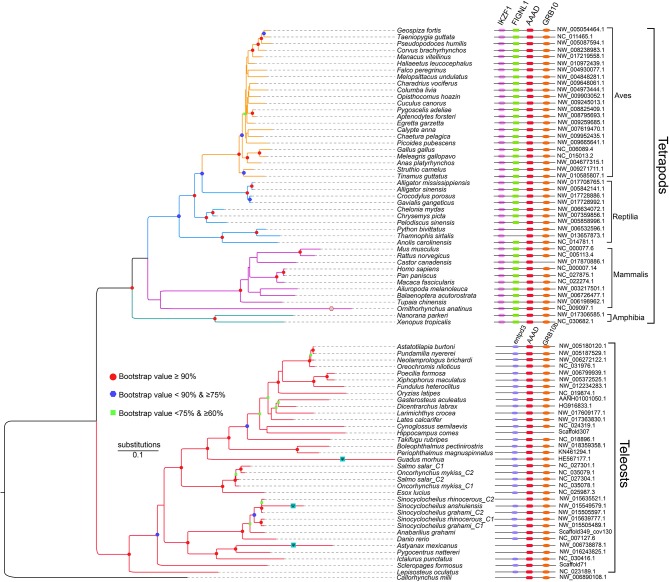
A phylogenetic tree based on 81 AAAD synteny sequences (adapted from Li et al., 2018). Shapes in the nodes represents bootstraps, and only the values over 60% are presented. In detail, green squares stand for bootstraps ≥60 and <75%; blue hexagons represent bootstraps ≥75 and <90%; red dots indicate bootstraps ≥90%.

**Figure 4:**
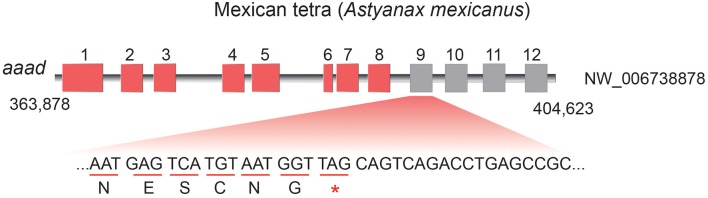
Premature stop in the *aaad* encoding region of Mexican tetra (*A. mexicanus*). 5-HT and melatonin synthesis may be blocked in this cavefish.

Unlike only one copy in diploid fishes, there are two copies of *aaad* gene possibly due to additional WGD in tetraploid teleosts. Interestingly, some animals with residency in darkness such as platypus (*Ornithorhynchus anatinus*), Mexican tetra (*Astyanax mexicanus*), and a *Sinocyclocheilus* cavefish (*S. anshuiensis*; Yang et al., 2016) display longer evolutionary branches in the phylogenetic topology (Figure 3) than other species. This reflects a fast evolution of the *aaad* genes in those species, suggesting a functional variation possibly due to the dark environments. In addition, we identified premature stop in the encoding region of *aaad* gene in the cave-restricted Mexican tetra (Figure 4), implying a possibility of weakening or disappearing rhythms in cavefishes (Yang et al., 2016).

### Aralkylamine N-Acetyltransferase (AANAT)

AANAT is the main rate-limiting enzyme for melatonin biosynthesis, converting 5-HT to NAS. The vertebrate-type AANAT evolved from a more primitive non-vertebrate-type (Falcón et al., 2014), which is widely known from organisms evolutionarily more ancient than Agnathans including amphioxus (Pavlicek et al., 2010). During the early evolution in vertebrates, *aanat* gene was duplicated and one of the paralogs apparently diverged to become the vertebrate-type AANAT. However, the non-vertebrate-type was lost from almost all vertebrate lineages, although the two forms coexist in certain chondrichthyes such as in the elephant shark *Callorhinchus milii* (Falcón et al., 2014). Before the divergence of Gnathostomes, duplication of the vertebrate-type AANAT happened, while only one copy was kept in tetrapods. However, in teleosts, the two copies (called *aanat1* and *aanat2*) were retained; subsequently, *aanat1* was reproduced once more to be divided into *aanat1a* and *aanat1b* (Li et al., 2016). During teleost evolution, these gene duplications and selective loss of certain isotype(s) potentially regulate 5-HT conversion and melatonin production.

AANAT1 is reported to be more specifically distributed in the retina and brain, while AANAT2 is mainly expressed in the pineal gland (Falcon et al., 2010). In our previous report (Li et al., 2016), we identified two important residues (130 and 153) are obviously differentiated between AANAT1 (F, V) and AANAT2 (C, L). According to the template of 1CJW (Hickman et al., 1999), we propose that the two sites may be involved in formation of a α-helix and a β-sheet, respectively. These structural differences also point to potentially different roles between AANAT1 and AANAT2.

In some vertebrates, low AANAT levels can also be measurable in other areas, such as gastrointestinal tract and skin (Coon and Klein, 2006; Velarde et al., 2010a). The diversity of *aanat* genes in fishes (*aanat1a, aanat1b*, and *aanat2*) is usually generated by WGD and gene loss (Li et al., 2016). During evolution in diverse environmental conditions, *aanat1* and *aanat2* were differentially expressed in various organs and played differential roles. Mainly due to the circadian rhythmic activity of AANAT, blood levels of melatonin increase at night and decrease during daytime. Our recent investigation (Li et al., 2016) indicates that bony fishes possess various isoforms of *aanat* genes, whereas other vertebrates have only a single form of AANAT. Two rounds of WGD in fishes are responsible for the classification of three isotypes of *aanat* (*aanat1a, aanat1b*, and *aanat2*); however, gene loss somehow resulted in absence of certain isotypes in certain special fishes, such as amphibious mudskippers. We have predicted loss of *aanat1a* in a more terrestrial mudskipper (*Periophthalmus magnuspinnatus*, PM), which was regarded to participate in terrestrial vision changes (You et al., 2014).

**Figure 5:**
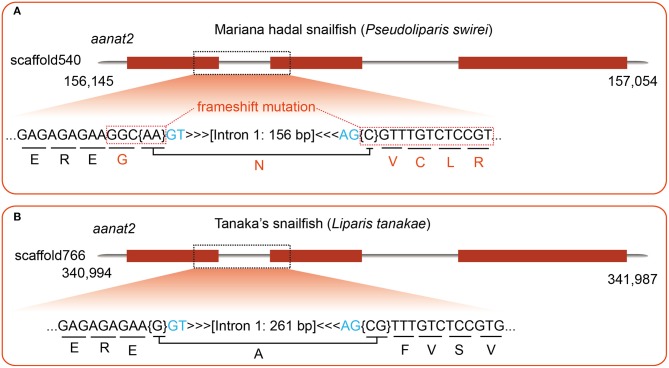
A detailed comparison of *aanat2* encoding regions between Mariana hadal sailfish (MHS; resident below 6,000 m) and its close relatives Tanaka's snailfish (TS; collected from shallow sea). **(A)** A frameshift insertion is identified in the MHS by genomic sequence alignment, using zebrafish *aanat2* as the query. **(B)** With the same query, however, the TS *aanat2* in the same region is found to be normal.

Recently, a draft genome of *Pseudoliparis swirei*, a deep-sea snailfish (Mariana hadal snailfish, MHS) with a routine residence below 6,000 m, was published (Wang et al., 2019). Meanwhile, the genome of its closed relative Tanaka's snailfish (*Liparis tanakae*, TS) from shallow sea was also available in the same report. Here, we performed a detailed comparison of *aanat2* gene structures between the MHS and the TS, and observe a frameshift insertion (C) in the MHS (Figure 5A) while its relative Tanaka's snailfish is normal (Figure 5B). Interestingly, the one-base insertion may lead to inactivity of AANAT2 in the MHS, hence consequent low levels of blood melatonin in the MHS are predictable. These are possibly related to the deep-sea darkness adaption, which is similar to our previously reported cave-restricted *Sinocyclocheilus anshuiensis* (Yang et al., 2016).

### N-Acetylserotonin Methyltransferase (ASMT)

ASMT, previously named as hydroxyindole-O-methyltransferase (HIOMT), is the ultimate enzyme for melatonin biosynthesis (Figure 1A). It is assumed to be responsible for seasonal variations in the melatonin secretion and vertebrate reproduction. It belongs to the methyltransferase superfamily with a wide identification in animals and plants.

In rice, there exist three isotypes of *asmt* (*asmt1, asmt2*, and *asmt3*); all of them could encode active ASMT, and overexpression of them could generate overproduction of melatonin (Kang et al., 2011; Park et al., 2013a,b). In mammals, the *asmt* gene is usually located on the X chromosome (Rodriguez et al., 1994; Wang et al., 2013). Resulting from alternative splicing of exons 6 and 7, human *asmt* possibly possesses three isotypes, of which one isoform catalyzes the traditional transference of a methyl group to produce melatonin; however, other two isoforms lost this enzyme activity (Botros et al., 2013). In fish genomes, in contrast to tetrapods, two *asmt* genes have been identified, possibly due to the putative TWGD event (Velarde et al., 2010a).

It was reported that the ASMT enzymes mainly exist in the retinae and pineal gland of European sea bass (Botros et al., 2013). ASMT2 was detected in several peripheral tissues, including liver and gut in teleosts (Paulin et al., 2015). The similar existence of melatonin synthesis in gut and liver of goldfish was demonstrated previously (Velarde et al., 2010a). Recent studies also suggested high expression of gut ASMT in zebrafish and rainbow trout (*Oncorhynchus mykiss*) (Khan et al., 2016; Muñoz-Pérez et al., 2016). Findings on a tropical carp (*Catla catla*) recently indicated that abundant *asmt* mRNAs were detectable in the gut, and the transcription levels of *asmt* in the brain displayed a significant negative correlation with cultivated water temperature (Sanjita Devi et al., 2016). Moreover, the involvement of brain melatonin in modulation of seasonal reproductive parameters through the putative hypothalamo-pituitary-gonadal (HPG) axis has been suggested by high expression levels of ASMT in the brain during the preparatory phase (Velarde et al., 2009, 2010b; Mukherjee and Maitra, 2015).

**Figure 6:**
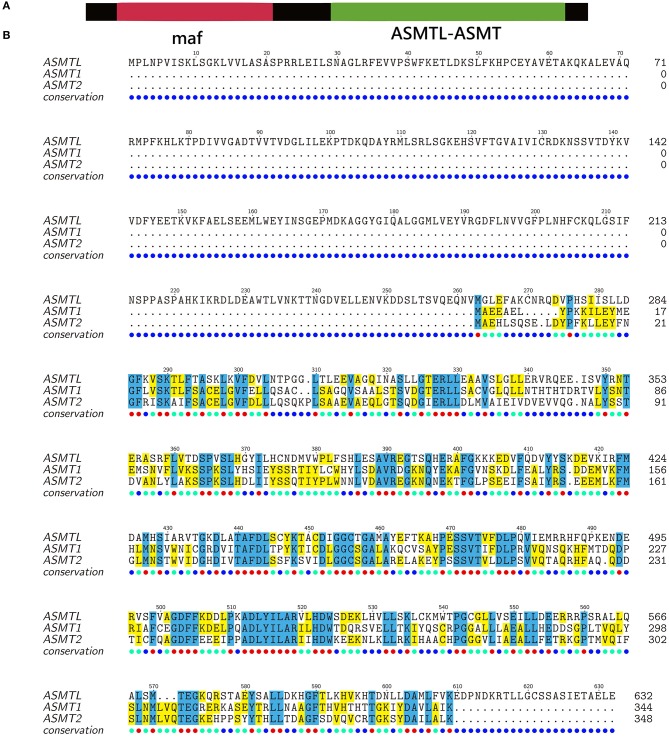
Alignment of ASMT protein sequences of zebrafish (modified from Zhang et al., 2017). **(A)** ASMTL is encoded by a fusion gene of maf (multicopy associated filamentation) and *asmt*. **(B)** The C-terminal of ASMTL is highly similar to ASMT1 and ASMT2.

However, a previous paper (Ried et al., 1998) reported acetylserotonin methytransferase-like (*asmtl*) genes in mammals, which is novel with an interesting homology to the putative *asmt* genes. In fact, human *asmtl* is a fusion gene of *maf* and *asmt* (similar to the zebrafish in Figure 6A). Through a comprehensive genomic investigation (Zhang et al., 2017), we also identified existence of ASMT1, ASMT2, and ASMTL in fishes (Figure 6B) and identified some variants in their alignments between ASMT1 and ASMT2. Two of the variants (E61 and Q243) were reported with reduced ASMT activity (Botros et al., 2013), thereby a functional divergence between ASMT1 and ASMT2 can be predicted. In addition, teleost-ASMTL is consistent with mammal-ASMTL (Figure 6A), formed by gene fusion (*maf* and *asmt*) and duplication events. In addition, our transcriptome data revealed that *asmt1* was preferentially transcribed in fish retinae and pineal gland, while *asmt2* and *asmtl* were mainly expressed in the fish peripheral tissues such as liver, gut, skin, and gonad (Zhang et al., 2017). These results imply that the functional roles of these ASMTs are different.

## Conclusions

Although the serotonin/melatonin biosynthesizing enzymes are shared by vertebrates, there are a lot of molecular differences between teleosts and tetrapods. More isotypes of enzymes in fishes are possibly related to the teleost-specific genome duplication event(s) and gene loss. Functional roles among different isotypes of the four biosynthesizing enzymes in serotonin and melatonin production are differentially various. Interestingly, loss of environmental light stimuli may cause gene loss or molecular mutations in many deep-sea and caved fishes, indicating that serotonin and melatonin biosynthesis may be originated from the rhythmic adaption to exogenous light/dark shift. Although more and more studies on mammals reveal detailed functional roles of the serotonin and melatonin biosynthesizing enzymes, corresponding functional identifications in fishes (with more isotypes) are still limited. In summary, we provide an overview of recent discoveries about molecular evolution of the four serotonin-related enzymes in vertebrates, with a focus on genomic differences between mammals and fishes.

## Author Contributions

QS and CQ conceived and designed the project and revised the manuscript. YLv and YLi performed the genomic investigations and wrote the manuscript. JL and CB participated in discussion and figure preparation.

### Conflict of Interest

The authors declare that the research was conducted in the absence of any commercial or financial relationships that could be construed as a potential conflict of interest.
